# End-to-end pipeline for automated heart failure diagnosis with clinical notes using SNOMED-CT

**DOI:** 10.1038/s41598-026-48771-1

**Published:** 2026-04-19

**Authors:** Fu-Sung Kim-Benjamin Tang, Marlo Verket, Dirk Müller-Wieland, Julia Brandts, Malte Jacobsen, Andreas Pütz, Katharina Marx-Schütt, Nikolaus Marx, Thomas Schmitz-Rode, Ioana Slabu

**Affiliations:** 1 Institute of Applied Medical Engineering, Helmholtz Institute, Medical Faculty, RWTH Aachen University, Aachen, Germany; 2 Department of Internal Medicine I, University Hospital Aachen, Aachen, Germany; 3 Institute of Medical Engineering, Helmut Schmidt University, Hamburg, Germany

**Keywords:** Cardiology, Information technology, Computer science, Diagnosis, Data processing, Machine learning, Predictive medicine

## Abstract

Diagnosis of heart failure is complex but crucial for patient outcomes and often hindered by the untapped potential of unstructured clinical notes. We introduce a novel end-to-end pipeline for heart failure diagnosis, leveraging electronic health records (EHR) and German clinical notes from 846 patients. Our pipeline synthesizes abbreviation disambiguation, translation of German clinical notes to English, medical entity linking to SNOMED-CT, and subsequent classification. The classification was performed using a Support Vector Machine (SVM) and compared against a fine-tuned medBERT.de neural baseline. We reduced the reliance on training data with zero-shot learning to address limitations with abbreviation disambiguation and entity linking approaches. Validation against benchmark datasets and cardiologists demonstrates high accuracy for real clinical use. Abbreviation disambiguation achieved an accuracy of up to 96.1%. Entity linking achieved competitive performance compared to state-of-the-art approaches on selected evaluation datasets. The SVM classification approach utilizing SNOMED-CT concepts and EHR data achieved an F1-score of 65.3%, on par with the medBERT.de neural baseline using clinical notes and EHR data. Despite challenges regarding limited language-specific resources and reference dataset availability for SNOMED-CT annotations in German, our pipeline demonstrates high potential for real-world clinical use and clinical decision support grounded in the standardized SNOMED-CT ontology.

## Introduction

 Artificial intelligence (AI) in clinical decision support systems (CDSS) can assist in diagnosing life-threatening diseases, including respiratory diseases or cardiovascular diseases, such as pneumonia, chronic obstructive pulmonary disorder, or heart failure^1–3^. Heart failure is a prevalent disease with high mortality rates in part due to frequent misdiagnosis and late-stage detection^4^. Timely diagnosis and early intervention are challenging yet essential for reducing mortality^5^. However, early diagnosis is hard to achieve due to limited patient data in early disease stages. Clinical notes containing up to 80% of patients’ healthcare data are available at early disease stages and can enable detection up to two years before the diagnosis^6,7^. However, clinical notes are underutilized in CDSS because information extraction from free text is complex due to semantic ambiguity and very resource-intensive when performed manually^8–10^.

Current heart failure diagnostic approaches are based on guidelines of the European Society of Cardiology (ESC) or rely on novel machine learning by supplementing electronic health records (EHR) with limited extracted information. At most, only a fraction of available patient information in clinical notes, such as individual mentions of selected keywords, is utilized due to challenges presented by semantic ambiguity^11,12^. Semantic ambiguity caused by abbreviations, synonyms, and contextual variations can be resolved through context-based abbreviation expansion and entity linking to standardized medical terminologies such as the Unified Medical Language System (UMLS) or SNOMED-CT. State-of-the-art approaches demonstrated high accuracy comparable to board-certified physicians when expanding abbreviations^13^, and high accuracy when linking entities to medical terminologies^14^.

However, current abbreviation expansion and entity linking approaches face problems with scalability as the number of abbreviations increases, generalizability to new abbreviations outside the training data, or availability of extensive manually curated training datasets^13,15,16^. Approaches leveraging zero-shot learning, such as MedCAT^14^ with its syntactic token-based entity linking algorithm, can link entities without required training data but face limitations when term order changes, novel synonyms are introduced, or paraphrased medical information arises. Moreover, existing research predominately focuses on English use cases, reducing transferability to use cases in other languages, such as German, with limited resources regarding datasets for model training and evaluation, terminologies for data standardization such as SNOMED-CT, and previous research to bridge the language barrier.

This study addresses the challenges of semantic ambiguity in clinical notes and the scarcity of annotated resources for automated heart failure diagnosis. We hypothesize that combining context-aware abbreviation disambiguation with semantic entity linking to standardized medical terminologies, in a zero-shot learning framework, enables accurate and comprehensive heart failure prediction based on standardized clinical information - without the need for large, annotated datasets.

**Fig. 1:**
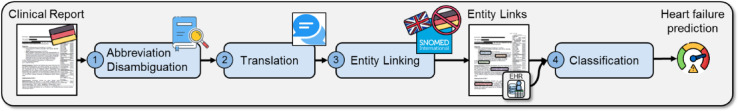
Pipeline overview. The end-to-end heart failure prediction pipeline is based on patient data in clinical notes and electronic health records (EHR). The pipeline consists of four steps for (1) abbreviation disambiguation, (2) translation of German clinical notes to English, (3) entity linking to standardize medical concepts to SNOMED-CT with the International SNOMED-CT version due to a lack of a complete German version and (4) classification to derive a heart failure prediction for a patient based on the extracted SNOMED-CT entity links and EHR data.

To test this hypothesis, we developed a novel end-to-end pipeline for heart failure diagnosis using AI and SNOMED-CT on standardized and unambiguous patient data extracted from German clinical notes and EHR. Our four-step pipeline leverages pre-trained language models for (1) zero-shot disambiguation of abbreviations, (2) the translation of German clinical notes to English to bridge the language barrier, and (3) the novel semantic zero-shot entity linking approach to link medical entities to SNOMED-CT and UMLS (Fig. 1). Finally, (4) the pipeline predicts heart failure based on data consisting of EHR data with SNOMED-CT concepts from the obtained entity links. The pipeline is adaptable via online learning, allowing incremental improvements based on physician feedback and new datasets, making it suitable for diverse clinical use cases. We evaluated the abbreviation disambiguation and entity linking steps of the pipeline with established English datasets to ensure comparability with other state-of-the-art methods. We also assessed the classification step on a German heart failure cohort, representing a concrete real-world clinical use case. For the translation step, we leverage a pre-trained and unmodified translation model and refer to its published performance. To our knowledge, our contribution presents the first heart failure prediction approach that leverages SNOMED-CT’s hierarchical structure to advance the field of AI-driven CDSS.

## Results

### Abbreviation disambiguation

We implemented context-based abbreviation disambiguation in the first step of our pipeline and evaluated the performance on the CASI dataset^17^ and the “WSRS Clinical Abbreviation” dataset^13^ regarding recall, precision, expansion accuracy and total accuracy (Eqs. (1),(2),(3),(4)).

### CASI dataset

The best performance on the CASI dataset was achieved with a threshold of 0.1, with a perfect recall of 100%, a precision of 60.1% and an expansion accuracy and total accuracy of 96.1% (Table 1).

**Table 1:** Abbreviation disambiguation performance on the CASI dataset.

Threshold	Recall in %	Precision in %	F1 score in %	Expansion accuracy in %	Total accuracy in %
0.1	**100.0**	60.1	**75.1**	**96.1**	**96.1**
0.2	99.7	63.8	77.8	94.8	94.5
0.3	95.5	70.7	81.2	87.2	83.3
0.4	79.0	75.3	77.1	68.8	54.4
0.5	49.1	77.7	60.2	41.6	20.4
0.6	20.0	79.5	32.0	17.6	3.5
0.7	4.8	81.6	9.1	4.3	0.2
0.8	0.5	90.3	1.0	0.4	0
0.9	0	**100**	0	0	0

Performance overview regarding detection recall, precision, expansion accuracy, and total accuracy of our abbreviation disambiguation on the CASI dataset with different context similarity thresholds. The maximum values are marked in bold.

### WSRS clinical abbreviation dataset

With the WSRS Clinical Abbreviation dataset, our approach yielded the highest total accuracy at a threshold of 0.1. Higher thresholds result in lower recall and with the highest threshold of 0.9, no abbreviations were disambiguated, resulting in the lowest performance (Table 2).

**Table 2:** Abbreviation disambiguation performance on the WSRS Clinical Abbreviation dataset.

Threshold	Recall in %	Precision in %	F1 score in %	Expansion accuracy in %	Total accuracy in %
0.1	**98.7**	71.9	83.2	65.4	**64.5**
0.2	98.4	73.9	84.4	65.5	64.4
0.3	94.3	84.5	**89.1**	66.0	62.3
0.4	75.2	93.1	83.2	69.7	52.4
0.5	38.8	97.7	55.5	76.1	29.5
0.6	11.7	**100**	20.9	85.9	10.0
0.7	2.3	**100**	4.4	**100**	2.3
0.8	0.5	**100**	0.9	**100**	0.5
0.9	0	0	0.0	0	0

Performance overview regarding recall, precision, expansion accuracy, and total accuracy of our abbreviation disambiguation on the WSRS Clinical Abbreviation dataset with different context similarity thresholds. The maximum values are marked in bold.

### Entity linking

We implemented entity linking in the third step of our pipeline to link medical concepts to SNOMED-CT or UMLS and evaluated the performance on the English datasets ShARe/CLEF 2014^18^ and MedMentions^19^ regarding recall, precision, and F1 score, and on the German DARIO dataset through an expert survey.

### English dataset evaluation

The highest recall without abbreviation disambiguation of 81.1% on ShARe/CLEF 2014 and 71.6% on MedMentions can be observed after the identification step for entity link candidates for the lowest threshold of 0.9, while an increasing threshold lead to a lower recall (Table 3). Abbreviation disambiguation leads to a higher recall with an increased recall of 3.6% on ShARe/CLEF 2014 but a slightly decreased recall of 0.1% on MedMentions. The precision after the candidate identification is very low with values ranging from 1% to 3%.

**Table 3:** Recall after the identification of entity link candidates.

Threshold	Without abbreviation disambiguation		With abbreviation disambiguation	
	ShARe/CLEF 2014 recall in %	MedMentions recall in %	ShARe/CLEF 2014 recall in %	MedMentions recall in %
0.90	**81.1**	**71.6**	**84.7**	**71.5**
0.91	80.6	71.0	84.1	70.9
0.92	79.8	70.6	83.6	70.5
0.93	79.2	69.9	82.9	69.8
0.94	78.4	69.3	82.1	69.1
0.95	78.0	68.6	81.6	68.5
0.96	77.0	67.9	80.5	67.8
0.97	76.5	67.1	80.0	67.0
0.98	75.4	66.1	78.9	66.0
0.99	74.4	65.0	77.8	64.9

Recall after the identification of entity link candidates with similarity thresholds of 0.9 to 0.99 on the ShARe/CLEF 2014 and MedMentions dataset with and without prior disambiguation of abbreviations. The maximum values are marked in bold.

The low precision motivated the subsequent entity link candidate selection step, which achieved the highest precision of 38.8% at a threshold of 0.99 on MedMentions and the highest precision of 80.2% at a threshold of 0.96 and 0.97 on ShARe/CLEF 2014 (Table 4). The highest F1 score was observed with 47.0% at threshold of 0.98 on MedMentions and with 76.6% at a threshold of 0.95 on ShARe/CLEF 2014.

**Table 4:** Entity linking performance without prior abbreviation disambiguation.

Threshold\dataset & metric	MedMentions (disorders only)			ShARe/CLEF 2014		
	Precision in %	Recall in %	F1 score in %	Precision in %	Recall in %	F1 score in %
0.9	32.4	59.0	0.418	60.3	64.9	62.5
0.91	34.1	59.5	0.434	76.6	**74.7**	75.6
0.92	34.8	59.8	0.440	77.3	74.4	75.8
0.93	35.9	60.4	45.0	77.4	74.2	75.7
0.94	36.9	**60.8**	45.9	79.1	73.8	76.4
0.95	34.9	54.8	42.6	79.9	73.6	**76.6**
0.96	37.7	60.7	46.6	**80.2**	73.0	76.5
0.97	38.3	44.7	41.3	**80.2**	72.8	76.3
0.98	38.4	60.6	**47.0**	80.0	72.1	75.8
0.99	**38.8**	55.7	45.8	80.0	71.7	75.6

Entity Linking performance regarding the recall, precision, and F1 score on the MedMentions and ShARe/CLEF 2014 datasets without prior abbreviation disambiguation and with different similarity thresholds in the entity link candidate selection step. The maximum values are marked in bold.

The entity linking performance of our pipeline with prior abbreviation disambiguation led to a slightly decreased F1 score by 0.2% of 46.8% on the MedMentions dataset at a threshold of 0.98 and an increased F1 score by 2.2% of 78.8% on the ShARe/CLEF dataset at a threshold of 0.95 (Tables 4 and 5).

**Table 5:** Entity linking performance with prior abbreviation disambiguation.

Threshold\dataset & metric	MedMentions (disorders only)			ShARe/CLEF 2014		
	Precision in %	Recall in %	F1 score in %	Precision in %	Recall in %	F1 score in %
0.9	26.7	46.6	33.9	60.8	67.4	63.9
0.91	35.1	60.4	44.4	77.2	**78.0**	77.6
0.92	35.9	**60.8**	45.1	78.1	77.9	78.0
0.93	35.6	59.9	44.7	78.3	77.6	77.9
0.94	36.6	60.4	45.6	79.8	77.3	78.5
0.95	36.3	55.9	44.0	80.5	77.1	**78.8**
0.96	37.6	60.3	46.3	80.8	76.4	78.5
0.97	38.2	60.3	46.7	**80.9**	76.2	78.5
0.98	38.3	60.2	**46.8**	80.7	75.5	78.0
0.99	**38.5**	44.6	41.3	80.7	75.1	77.8

Entity Linking performance regarding recall, precision, and F1 score on the MedMentions and ShARe/CLEF 2014 datasets with prior abbreviation disambiguation and with different similarity thresholds t in the entity link candidate selection step. The maximum values are marked in bold.

### German dataset evaluation

**Fig. 2:**
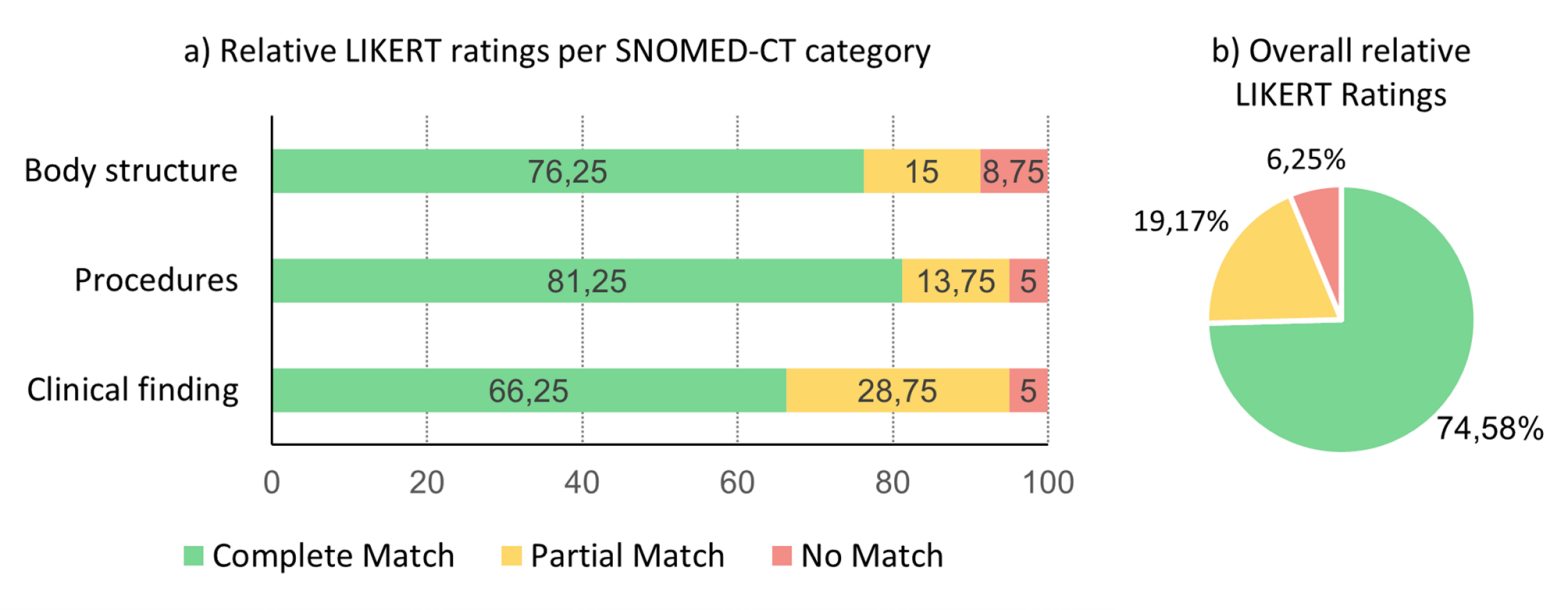
Expert survey results. Results of the expert survey conducted with three cardiologists who rated 40 entity links with concepts of the SNOMED-CT categories “Body structure”, “Procedures” and “Clinical finding” respectively on a 3-point LIKERT scale to capture the goodness of the match through the options “Complete match”, “Partial Match” and “No Match”. Relative LIKERT ratings per SNOMED-CT category are shown on the bar plots (**a**) while the overall relative LIKERT ratings are shown in the pie chart (**b**).

We conducted an expert survey to evaluate the entity linking performance on the German DARIO dataset with three cardiologists, who rated the goodness of entity links on a 3-Point LIKERT scale. The cardiologists found that most entity links were a complete match, where entity links of the category “Procedures” had the highest number of complete matches, and entity links of the category “Clinical findings” had the lowest number of complete matches (Fig. 2). The agreement between cardiologists rating the entity links per SNOMED-CT category calculated through Cohen’s Kappa score indicated low agreement with scores of 0.16 for “Body structures”, 0.23 for “Procedures” and 0.43 for “Clinical finding”.

### Heart failure classification

We implemented heart failure classification of patients in the fourth step of our pipeline and evaluated the performance through the recall, precision and F1 score.

The SVM classifier using patient information from EHR and SNOMED-CT concepts from entity links yielded the highest F1 performance of 65.3% alongside the late fusion classifier using the fine-tuned medBERT.de model. Combining EHR with SNOMED-CT concepts from entity links improved the F1 score for the SVM by 8.2% compared to the classifier using only patient information from EHR and by 1.6% compared to the classifier using only SNOMED-CT concepts from entity links (Table 6). For the fine-tuned medBERT.de model, the late fusion with EHR records improved the F1 score of 63.8% by 1.5%.

**Table 6:** Performance comparison of heart failure classifiers using different patient data types.

Classifier trained with	Precision in %	Recall in %	F1 in %
Patient information from EHR (SVM)	58.6	57.4	57.1
SNOMED-CT concepts from entity links (SVM)	63.6	65.0	63.7
Patient information from EHR + SNOMED-CT concepts from entity links (SVM)	64.9	**66.4**	**65.3**
Clinical notes (fine-tuned medBERT.de)	64.9	64.3	63.8
Clinical notes + Patient information from EHR (late fusion with fine-tuned medBERT.de)	**66.2**	65.2	**65.3**

Comparison of the SVM classifier (only EHR, only SNOMED-CT links, ensemble EHR and SNOMED-CT links combined) against the neural baselines (medBERT.de and multimodal late fusion). The performance was evaluated through 5-fold cross validation on the whole patient data using the metrics precision, recall and F1 score. The maximum values for each metric are marked in bold.

**Fig. 3:**
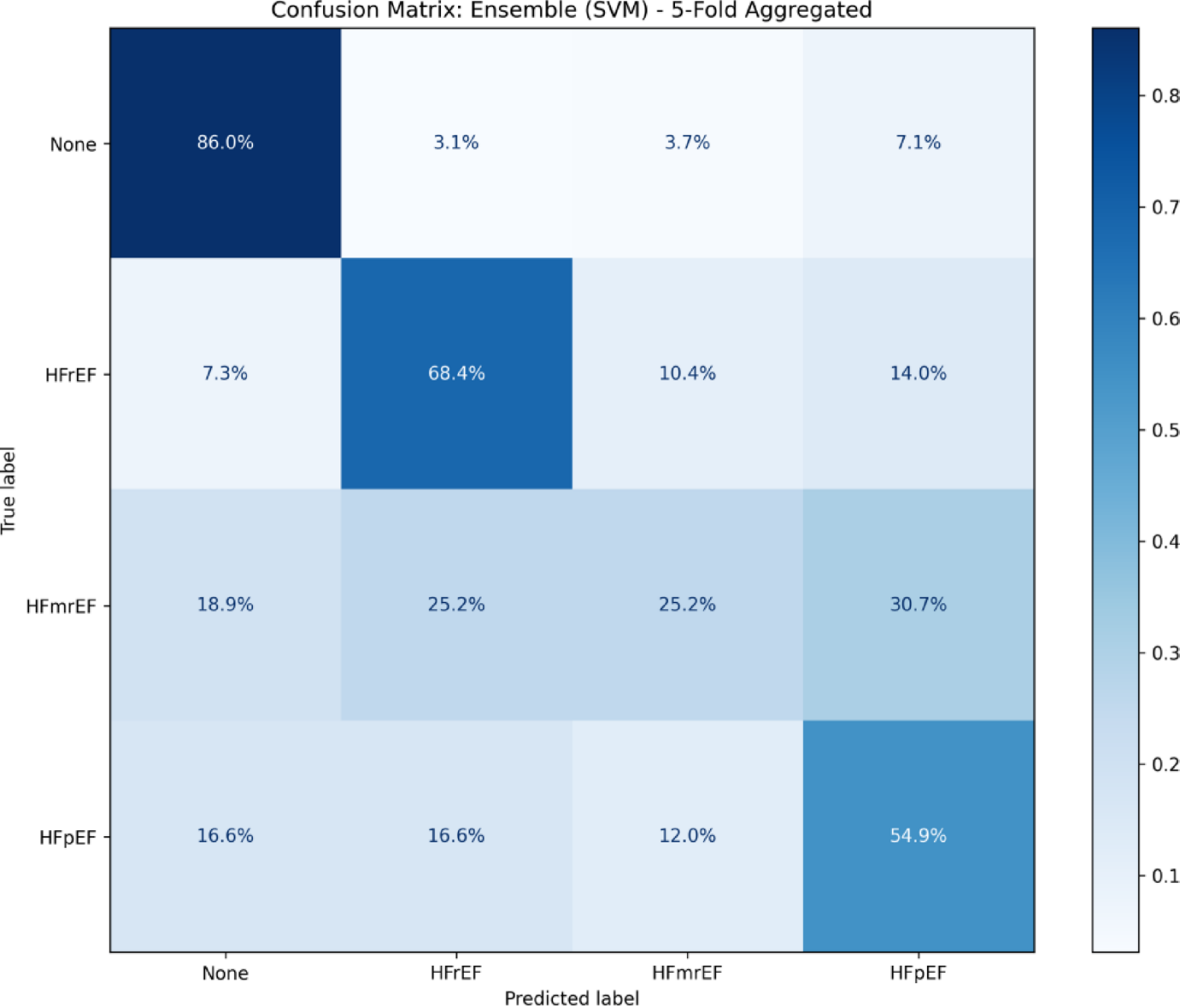
Confusion matrix of heart failure classification. Confusion matrix for the classification accuracy of the heart failure diagnosis of patients without heart failure (No HF) and with heart failure where the ejection fraction is: reduced (HFrEF), mildly reduced (HFmrEF), and preserved (HFpEF).

Moreover, we analyzed the confusion matrix of the SVM classifier trained on both EHR data and entity links to evaluate its performance in classifying patients into the four heart failure diagnosis groups. The confusion matrix depicts the accuracy of the classifications regarding the actual diagnosis groups of patients with the predicted diagnosis groups of the classifier (Fig. 3). The highest accuracy can be observed for the diagnosis group of having no heart failure with 86.0% followed by HFrEF with 68.4%, while HFpEF classifications yield an accuracy of 54.9% and the lowest performance by far can be observed for the diagnosis group HFmrEF with 25.2%. Patients suffering from HFmrEF are predominately misclassified as HFpEF followed by HFrEF, indicating possibly strong overlaps regarding clinical findings mentioned in the clinical notes or stored in the EHR. Patients suffering from HFpEF were mostly misclassified as having no heart failure or suffering from HFrEF.

## Discussion

In this study, we developed an end-to-end pipeline to assist in heart failure diagnosis that leverages retrospective data of electronic health records and German clinical notes from 846 patients with and without heart failure and demonstrated its predictive effectiveness.

Our multi-step pipeline synthetizes abbreviation disambiguation, translation of German text to English and free-text entity linking to SNOMED-CT or UMLS to diagnose heart failure based on patient data consisting of entity links and EHR. A key feature is the zero-shot learning approach for abbreviation disambiguation and entity linking, eliminating the need for annotated training data. Our pipeline achieves high diagnostic accuracy with an F1-score of up to 65.3% and has the potential to be used as part of a clinical decision support system for physicians to streamline and enhance the early diagnosis process.

### Abbreviation disambiguation

The first step of our pipeline disambiguates abbreviations to reduce semantic ambiguity, which is common in clinical language^20^. The high prevalence of unique abbreviations in clinical notes complicates effective communication and underlines the need for disambiguation in clinical decision support^13,15,21,22^. Recent abbreviation disambiguation approaches leveraging language models achieve accuracy similar to board-certified physicians, but require manually curated training datasets^13,15,16^. To increase the efficiency, we enabled zero-shot disambiguation of over thousands of abbreviations in our pipeline without training data, by creating an English and German abbreviation dictionary with context samples from clinical notes of the MIMIC-III and GGPONC datasets respectively and utilized pre-trained language models to expand abbreviations in our datasets based on surrounding context^23,24^. Our approach achieved a recall of 100% and a total accuracy of 96.1% on the ASI dataset, outperforming the current state-of-the-art performance^13^. Measuring the precision does not provide a reliable precision indicator in the CASI dataset, since snippets in the CASI dataset are only annotated with the expansion of one occurring abbreviation, regardless of the presence of multiple abbreviations^13^. This means a sentence could contain multiple correctly detected abbreviations, but with only one labeled ground truth annotation, further correct abbreviation detections would degrade the reported precision.

Hence to evaluate the precision and overall performance of our implemented abbreviation disambiguation further, we leveraged the WSRS Clinical Abbreviation dataset, which is particularly challenging due to its broad scope with over 380 unique abbreviations in 302 snippets with limited occurrence in reference datasets such as MIMIC-III. Consequently, few context samples for 100 rarely occurring abbreviations and no context samples for 59 abbreviations could be retrieved from MIMIC-III. Our findings align with previous research that indicates a performance decline with fewer context samples per abbreviation, especially for niche or rarely occurring terms^14^. Future work could utilize datasets besides MIMIC-III, such as biomedical publications from PubMed to improve context coverage for rare abbreviations. Nevertheless, our fairly high zero-shot abbreviation disambiguation performance of 64.5% is promising for languages lacking suitable training datasets. Other existing zero-shot abbreviation disambiguation approaches achieve a higher 73% accuracy on the English WSRS Clinical Abbreviation dataset, outperforming our approach, but severely underperform when disambiguating German abbreviations^22^. While resources such as annotated datasets or expert availability were limited in this study, future work could explore the performance of our abbreviation disambiguation approach on German clinical notes.

### Translating clinical notes

The second step of our pipeline translates German clinical notes to English, which is necessary to overcome the language barrier when linking medical information in German to the international SNOMED-CT version^25,26^. An exploratory study addressed this language barrier similarly by translating German clinical notes to English and linking them to SNOMED-CT through MedCAT) with an F1-score of 0.5, highlighting the need for improvement^27^. Other studies translated SNOMED-CT to German through machine translation against SNOMED International’s advice, as semantic equivalence of SNOMED-CT terms regarding hierarchical positions is not considered^28–30^. Moreover, the strength of context-based machine translations cannot be leveraged with context-free term-based translations of SNOMED-CT, which is underlined by lacking accuracy during translation of clinical terms^31^.

### Entity linking to medical concepts

The third step of our pipeline links entities in clinical notes to UMLS or SNOMED-CT concepts, which is essential to resolve semantic ambiguity^32^ and to standardize medical information for improved patient healthcare^33^.

We chose SNOMED-CT as the primary terminology for entity linking due to its comprehensive coverage of clinical concepts relevant to heart failure, including specific subtypes and associated findings. Unlike ICD, which is optimized for billing and epidemiology, and LOINC, which focuses on laboratory and observation data, SNOMED-CT enables detailed and standardized broad representation of clinical information. UMLS serves as the secondary terminology in this study, as it aggregates a wide range of biomedical vocabularies, including SNOMED-CT, and thus expands the coverage of possible clinical concepts. However, UMLS lacks the explicit hierarchical relationships and logical relations between concepts that SNOMED-CT provides, which limits its expressiveness for clinical reasoning. By having the capability to leverage both SNOMED-CT and UMLS, our pipeline maximizes concept coverage for different use cases for clinical information extraction.

Automating the entity linking process is crucial, as manual entity linking requires scarcely available expertise and is associated with potential bias^34^. Novel medical entity linking approaches such as MedCAT combine syntactic term-based candidate identification with context embeddings to achieve state-of-the-art performance on the established benchmark datasets MedMentions and ShARe/CLEF 2014^14^. MedCAT extracted medical information from English clinical notes to assist with heart failure diagnosis in previous studies^12,35^, but we noticed limitations when testing MedCAT with our translated German clinical notes. The syntactic term-based algorithm for the concept candidate identification does not consider semantics in texts, limiting MedCAT’s recall for instance due to syntactic variants. Our pipeline’s novel entity linking approach with the two steps “candidate identification” and “candidate selection” was benchmarked with and without prior abbreviation disambiguation on the ShARe/CLEF 2014 and MedMentions datasets. The abbreviation disambiguation led to improved performance in our pipeline, with increased performance of up to 2.2% on the ShARe/CLEF 2014 dataset, while having a negligible performance degradation of 0.2% on the MedMentions dataset (Tables 4 and 5). The candidate identification step achieved a high recall of up to 81.1% but yielded a low precision of 1–3% (Table 3), motivating the subsequent candidate selection for improved precision. Through the candidate selection step our pipeline achieved a higher performance compared to MedCAT regarding the F1-score on ShARe/CLEF 2014 by 5%, but a 2.7% lower F1 score on MedMentions^14^. Higher performance could stem from improvements through the semantic candidate identification step, which leverages semantic language models pre-trained on semantic similarity tasks, resulting in a high recall. Additionally, we retrieve the top 10 candidate concepts for each n-gram, to reduce the risk of excluding correct concepts due to marginally differing similarity scores. Incorrect candidates can then be iteratively filtered out during the online learning process. To support linking to concepts with long clinical phrases, we configured n-grams with lengths ranging from 1 to 30, enabling alignment with complex SNOMED CT concepts - such as “*Coronary artery bypass grafting using free right internal thoracic artery graft from left internal thoracic artery to diagonal branch of anterior descending branch of left coronary artery*” with the identifier 1,156,983,007, which consists of 27 words.

Further, the abbreviation disambiguation improved performance, which aligns with research highlighting the need for abbreviation disambiguation for increased recall^36^. Lower performance could be attributed to MedCAT’s usage of proprietary data from UK university hospitals and expert feedback as well as possibly differing evaluation methods^37^.

The entity linking performance on our German use case dataset DARIO was evaluated with three cardiologists through an exploratory expert survey, similar to previous evaluations with expert surveys in the domain of ophthalmology^38^ and across numerous healthcare terminologies^39^. Unlike other available German datasets containing clinical notes, such as GGPONC^24^ or the cardiovascular-specific CARDIO: DE dataset^40^, DARIO uniquely includes a confirmed heart failure diagnosis – or documented absence in healthy individuals – for each patient’s clinical note, enabling clinically grounded evaluation of our pipeline. The expert survey results indicate a high performance on German medical free text with 74% correctly linked entities, especially compared to studies conducted for use cases in other languages yielding lower accuracy of 54.7%^41^.

A further investigation on the rather low inter-rater agreement with a Cohen’s Kappa of 0.16 for entity links in the category “Body Structures” revealed that the experts only fully disagreed on three out of forty linked concepts with opposite ratings and fully agreed on 25 out of 40 linked concepts, resulting in an absolute agreement of 62.5%. The reason for the low Cohen’s Kappa despite a rather high absolute agreement is the Kappa Paradox, which is caused due to the underlying dataset imbalance, with an overrepresentation of correctly linked concepts (positive samples) in the survey and a lower number of incorrectly linked concepts (negative samples)^42^.

### Heart failure classification

The fourth step of our pipeline classifies patients into four groups: no heart failure or one of the three heart failure subtypes defined by the left ventricular ejection fraction: reduced (HFrEF), mildly reduced (HFmrEF), and preserved (HFpEF). HFmrEF and HFpEF are especially challenging to diagnose due to initially unspecific symptoms and heterogeneous phenotypes in patients^5^. Established risk assessment scores, such as H2FPEF or HFA-PEFF, guide the diagnosis with defined clinical variables but are rarely used in clinical practice due to complexity and time constraints^43^. Both scores require extensive patient data, typically obtained through echocardiography or biomarker tests when heart failure is suspected in late disease stages^43,44^. Existing approaches leverage data available in early disease stages such as information from clinical notes but only to supplement missing values in EHR, such as the left ventricular ejection fraction and the comprehensive information in clinical notes remains underutilized due to extraction challenges caused by semantic ambiguity.

Our pipeline’s classification step combines information from clinical notes standardized with SNOMED-CT alongside available EHR data. The combined approach achieved an F1-score of 65.3%, improving upon a classifier trained only on the EHR data by 8.2% or a classifier trained only on entity link data by 1.6% (Table 6). We use a Support Vector Machine (SVM) for the classification, since SVMs are well-suited for sparse data, as they are robust to high dimensionality and can effectively handle sparse input without overfitting, particularly when available training data is limited^45^. The input feature vectors for our classification step comprise weighted SNOMED-CT concepts with structured EHR data, and are inherently high-dimensional and sparse, as patients are represented by a large set of possible clinical concepts, most of which are absent in any individual record.

Our classification approach incorporates entity link data analogous to related work^46^, but uses a more nuanced weighting scheme through term frequency – inverted document frequency (TF-IDF) weighting as opposed to frequency counts. However, diagnosing HFpEF remains challenging as observed through the confusion matrix, which aligns with known diagnostic challenges regarding the overlap of symptoms and clinical presentations with non-cardiac conditions contributing to HFpEF misclassification^47^. We compared our combined SVM approach with a neural baseline using medBERT.de as a fine-tuned model on the German medical domain and discovered the same end-to-end performance when diagnosing heart failure in patients using clinical notes and respective EHR.

Both approaches are resource-intensive to compute semantic embeddings and leverage clinical domain knowledge, either through ontology knowledge via SNOMED-CT or knowledge through fine-tuning on medical documents. However, the SVM approach using TF-IDF weighted SNOMED-CT concepts allows for a more transparent feature importance analysis to pinpoint which medical concepts drive a diagnosis for individual patients or even patient cohorts. Moreover, by grounding clinical notes in an ontology, such as SNOMED-CT, our pipeline enables granular patient cohort definition and advanced data mining that raw text embeddings through models like medBERT.de cannot easily support. Regarding international interoperability for cross-border clinical research, mapping to international SNOMED-CT concepts facilitates the integration of multi-lingual clinical reports and ensures the model’s findings are mapped to globally recognized medical standards.

### Error analysis

The lower performance of the medBERT.de model compared to our SVM-based pipeline may be attributed to the transformer’s fixed sequence length limitation (512 tokens). Clinical notes of patients in the DARIO cohort range from one to up to five pages, therefore frequently exceeding this fixed token length. Consequently, the BERT model likely processed only the initial sections or first page of the notes, potentially omitting critical diagnostic indicators located in later sections. In contrast, our TF-IDF/SVM approach considers the entire document by aggregating all extracted SNOMED-CT concepts, providing a more comprehensive patient representation.

The SVM classifier faces challenges when encountering unseen concepts in the test set, as they are not part of the TF-IDF matrix created from the training dataset, leading further to higher dimensionality and sparsity. However, we mitigate this challenge by traversing the SNOMED-CT hierarchy to include all ancestor concepts, so that the model can generalize from specific, rare terms to more frequent broader clinical categories. Thus, while the SVM lacks the nuanced contextual understanding of neural models, it can benefit from the vast ontological medical structure of SNOMED-CT.

Moreover, the high misclassification of HFmrEF patients as patients having HFrEF or HFpEF highlights the diagnostic complexity and clinical ambiguity of this subtype, considered an intermediate HF type between HFpEF and HFrEF, where symptoms often overlap with both other subtypes^11^. This suggests that while entity linking captures the existence of medical concepts, capturing the associated finer details such as severity and relevant context of symptoms needs to be explored further in the future to allow better discrimination between closely related subtypes.

However, regarding the medical entity links, we noticed a discrepancy between our entity linking results and the ShARe/CLEF 2014 gold standard annotations, which stems from the underlying annotation guidelines regarding negated concepts. While the guidelines suggest linking ‘no pain’ to the base concept ‘pain’ with a separate negation modifier, our pipeline - consistent with other state-of-the-art clinical NLP tools like MedCAT - retains the ability to link directly to specific pre-coordinated negated concepts in SNOMED-CT. To ensure a fair and direct comparison with the MedCAT baseline, we maintained this unified linking approach. This choice prioritizes the semantic specificity required for downstream heart failure classification, where pre-coordinated concepts often carry distinct clinical weight.

Another error source for incorrect entity links are disjoint spans for disorders, for example “a tumor was found in the left ovary”, where the words “tumor” and “ovary” should be linked as a disjoint span to the concept “ovarian neoplasm”. While our current entity linking implementation does not support disjoint spans, future work could leverage post-coordinated expressions to combine multiple medical concepts with relations to form more comprehensive concepts. Lastly, another error source stems from different concepts being identified by the same name, e.g., the SNOMED-CT concept “cell” can either refer to the “Cell structure” in the category “body structure” or “Cell (qualifier value)” as a unit of biological measurement. We address this challenge through the collection of positive and negative concept samples for unique concepts coupled with online-learning, but benefits of this implementation only become more apparent with larger datasets with recurring medical concept annotations.

When evaluating the correctness of our German medical entity links, we encountered low IAA as measured through Cohen’s Kappa score during our expert survey when evaluating entity links as low as 0.16 for “Body structure” concepts and with a maximum agreement of 0.43 for “Clinical finding” concepts (Fig. 2). While the low score of 0.16 stems from the Kappa Paradox, the overall rather low IAA highlights that linking medical entities to medical terminologies poses a frequently error-prone process for annotators leading to low Inter-Annotator Agreement (IAA)^38,48^. One possible reasons for low IAA are human issues regarding lacking medical knowledge, difficulties resolving abbreviations, lacking time, or lacking motivation in the entity linking process^49^. Another possible reason for low IAA is that multiple different yet semantically highly similar concepts in the target terminologies could be suitable for ground truth annotations^50^.

### Limitations

The expert survey for the entity linking results primarily measured the observed agreement between the cardiologists and the automated pipeline, effectively evaluating the system’s precision. Due to resource constraints, recall could not be assessed, as this would have required an exhaustive, independent manual annotation of the clinical notes by clinicians.

One notable limitation in the classification step is the usage of discharge summaries of the patients, which are typically generated post-diagnosis and may contain explicit references to heart failure. This introduces a risk of data leakage, as the model may learn to recognize diagnostic statements rather than infer patterns from earlier clinical cues, potentially inflating performance. Although a manual keyword review did not detect explicit heart failure diagnoses in the notes, late-stage indicators of the disease may still be present and contribute to leakage. Hence, we also reviewed the most frequently occurring linked concepts. Several cardiology-specific medical concepts, such as “Normal left ventricular systolic function”, “Depression of left ventricular systolic function” or “Stent placement” were retrieved by the entity linking pipeline and are used for the classification. However, these concepts also reflect medical information based on which cardiologists need to derive a non-trivial diagnosis. For instance, depending on the context, a normal left ventricular systolic function can indicate a healthy patient or a patient suffering from heart failure with preserved ejection fraction (HFpEF), whereas a depression of left ventricular systolic function could indicate heart failure with a reduced or mildly reduced ejection fraction (HFrEF, HFmrEF). While only discharge summaries of our patient cohort were available to evaluate the proof-of-concept for our pipeline, future work should assess and evaluate the pipeline with clinical routine notes at early disease stages.

### Outlook

The implementation and evaluation of our comprehensive heart failure diagnosis pipeline has provided a valuable foundation for further refinement and future research directions. Future research could improve classification performance with more precise representation of patient information through negation detection of medical concepts^51^ and the combination of multiple SNOMED-CT concepts through post-coordinated expressions to enable encoding of information not covered by existing concepts in SNOMED-CT^36^. Current approaches achieve post-coordination in SNOMED-CT through concept relation identification^52^, dependency parsing^53^ or knowledge graph embeddings^54^. Combining post-coordinated expressions with convolutional neural graph networks could then improve classification performances by processing patients represented through SNOMED-CT graphs instead of the currently used weighted SNOMED-CT concept lists for each patient^54,55^.

Besides focusing on the implementation of classification approaches, future research should also focus on the evaluation of developed approaches through further datasets and evaluation methods. The usage of additional datasets with SNOMED-CT annotations could lead to more representative results stemming from larger datasets covering multiple SNOMED-CT categories, such as the dataset from the SNOMED-CT Entity Linking Challenge sponsored by SNOMED International, once the respective test dataset of the challenge becomes publicly available^56^. While several English datasets exist for the evaluation of entity link approaches, datasets in German are lacking. The German datasets GGPONC^24^ and CARDIO: DE^40^ are promising resources, but do not link mentions to controlled terminologies such as SNOMED-CT or UMLS.

In conclusion, we developed and evaluated a comprehensive end-to-end pipeline for heart failure prediction from electronic health records and clinical notes. Our pipeline integrates abbreviation disambiguation, entity linking and classification, using zero-shot learning that reduces the need for training data in use cases with scarce or non-existent labeled training data. The pipeline achieves high performance when disambiguating abbreviations and linking free-text medical concepts to SNOMED-CT or UMLS and demonstrates its potential for application across diverse healthcare settings, particularly those with limited annotated resources in non-English use cases facing language barriers. Our findings demonstrate that an SVM-based classification approach leveraging SNOMED-CT concepts performs on par with state-of-the-art neural baselines like medBERT.de, while offering higher potential for interpretability, interoperability, and robustness to variable document lengths. Next steps should include the evaluation of our end-to-end pipeline to allow for a transfer into a clinical decision support system product and subsequently the application in clinical practice. Our pipeline enables precision medicine by compiling clinical patient information into SNOMED-CT, allowing patient and disease cluster analysis for targeted therapies. Moreover, the integration of explanation methods such as SHAP^57^ can promote transparency to increase trust by physicians and improve performance through a feedback-loop based on explanation insights. Our findings pave the way for future non-English medical use cases to not only diagnose heart failure early on for early therapy and reduced mortality but also to provide clinical decision support with the diagnosis of other diseases.

## Materials and methods

We implemented our heart failure prediction pipeline in Python and incorporated publicly available language models from Hugging Face (https://huggingface.co/models). Our pipeline required GPU resources for extensive computations with language models, which were performed on the High-Performance Cluster of the RWTH Aachen University with access to GPU computing resources powered by the NVIDIA H100 Tensor-Core-GPU.

Our heart failure prediction pipeline consists of four steps: (1) medical abbreviation disambiguation, (2) translation of German clinical notes to English, (3) medical entity linking of clinical notes to SNOMED-CT concepts, and (4) classification of patients based on their EHR and entity link data to diagnose heart failure.

### Disambiguating medical abbreviations

Abbreviation disambiguation approaches use abbreviation dictionaries containing medical abbreviations and their expansions. Our approach disambiguates abbreviations in English and German clinical notes with two abbreviation dictionaries, since medical abbreviations are language-dependent. For English clinical notes, we compiled a comprehensive abbreviation-expansion dictionary consisting of abbreviations from three different sources.

1. An openly available, manually curated English medical abbreviation-expansion dictionary from existing research, covering five knowledge sources with a total of 3758 unique abbreviations and 5794 unique abbreviation–expansion pairs^13^. This dataset was created using web-scale reverse substitution (WSRS), which substitutes medical abbreviations with their expansions in web-derived sentences, facilitating clinical abbreviation detection and expansion. For simplicity, this dataset is identified as WSRS Clinical Abbreviation dataset.
2. The Clinical Abbreviation Sense Inventory (CASI) dataset^17^, which supplements the WSRS dataset with additional abbreviation-expansion pairs.
3. Abbreviation-expansion pairs from the UMLS, which were extracted automatically by analyzing UMLS terms containing hyphens. For instance, in the abbreviation “AP” with the expansion “abdominal pain” in the UMLS term “AP – abdominal pain” was identified since the letters of the abbreviation matched the first letters of the term “abdominal pain” after the hyphen.

For the abbreviation disambiguation in German clinical notes, we supplemented 110 abbreviations provided by the Department of Internal Medicine I at the University Hospital Aachen with 1757 abbreviations from the medical association of Mecklenburg Vorpommern, which were aggregated for the competence training of physicians^58^.

To enable context-dependent disambiguation of abbreviations in our pipeline, we collected 30 context samples for each abbreviation, including surrounding context from English clinical notes in the established MIMIC-III dataset^23^. The number of 30 samples was chosen based on findings in previous research on effective context-based disambiguation^14^. For the disambiguation of German abbreviations, we aggregated 30 context samples from the openly available GGPONC dataset^24^, which represents one of the largest German datasets with medical documents based on clinical practice guidelines for oncology.

**Fig. 4:**
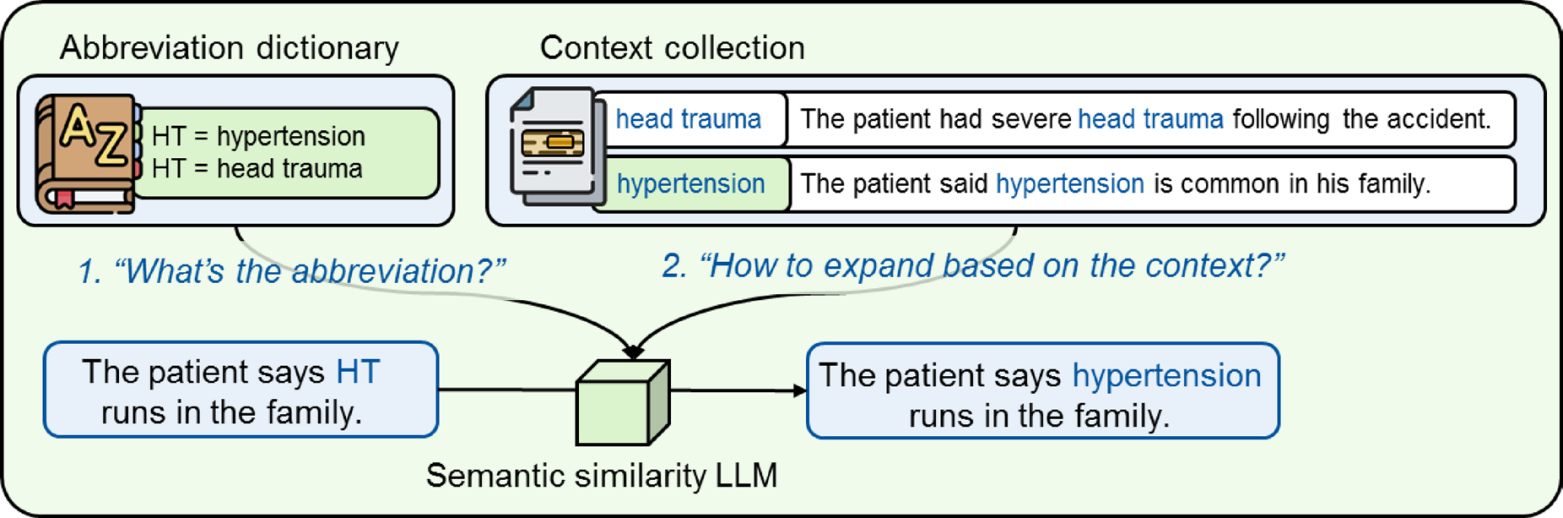
Overview of the abbreviation disambiguation approach. Our zero-shot abbreviation disambiguation approach detects abbreviations in clinical notes through pattern matching in the first step and expands abbreviations in the second text through context comparison. In this example, the first step of our approach identifies the abbreviation “HT” through pattern matching in the input phrase and finds the expansion candidates “hypertension” and “head trauma” through the provided abbreviation dictionary. In the second step, context samples for both expansion candidates are compared regarding semantic similarity through a language model with the context provided through the input phrase to select the expansion “hypertension” with the most similar context.

The abbreviation disambiguation in our approach involves two steps: first, identifying an abbreviation through common pattern matching based on known abbreviations in our abbreviation-expansion dictionary, and second, expanding the abbreviation based on its surrounding context (Fig. 4). To disambiguate the abbreviation, the surrounding context of the abbreviation in the sentence is compared by a language model pre-trained on semantic similarity (SS-LM) with a context collection for possible expansions of the abbreviation, following prior research^59^.

We use the pre-trained SS-LM *BioLORD*^60^ for English text and *paraphrase-multilingual-MiniLM-L12-v2*^61^ for German text. *BioLORD* was specifically trained to generate representations of clinical sentences and biomedical concepts from UMLS and SNOMED-CT, outperforming other state-of-the-art models in tasks such as semantic textual similarity as computed through cosine similarity, biomedical concept representation and biomedical concept name normalization^60^.

The SS-LM computes the similarity between each context sample for a given expansion candidate and the current text snippet, for instance, a sentence. The expansion candidate with the highest similarity is selected. We apply a threshold filter of 0.3 based on previous research to only expand an abbreviation, if the context has a cosine similarity of 0.3 or above, to avoid expansions when no similar context justifies them^14^.

We measured the abbreviation disambiguation performance through recall, precision, expansion accuracy and total accuracy. Recall reflects the proportion of correctly retrieved ground truth annotations, for example, abbreviations or concept identifiers with corresponding spans, and is defined as

1 $$\:Recall\:=\frac{True\:positives}{True\:positives+False\:negatives}$$.

The precision indicates how many of the retrieved items are correct and is defined as

2 $$\:Precision\:=\frac{True\:positives}{True\:positives+False\:positives}$$.

The expansion accuracy is defined as

3 $$\:Expansion\:accuracy=\left(\frac{\mathrm{C}\mathrm{o}\mathrm{r}\mathrm{r}\mathrm{e}\mathrm{c}\mathrm{t}\:\mathrm{e}\mathrm{x}\mathrm{p}\mathrm{a}\mathrm{n}\mathrm{s}\mathrm{i}\mathrm{o}\mathrm{n}}{\mathrm{C}\mathrm{o}\mathrm{r}\mathrm{r}\mathrm{e}\mathrm{c}\mathrm{t}\:\mathrm{e}\mathrm{x}\mathrm{p}\mathrm{a}\mathrm{n}\mathrm{s}\mathrm{i}\mathrm{o}\mathrm{n}+Incorrect\:Expansion}\right)$$

and the total accuracy defined as

4 $$\:Total\:accuracy=\left(Recall\cdot\:Expansion\:accuracy\right),$$

to ensure comparability with previous research^13^. Moreover, the harmonic mean of recall and precision forms the F1 score and is defined as:

5 $$\:F1\_Score=2\left(\frac{Precision\:\cdot\:\:Recall}{Precision+Recall}\right).$$

**Fig. 5:**
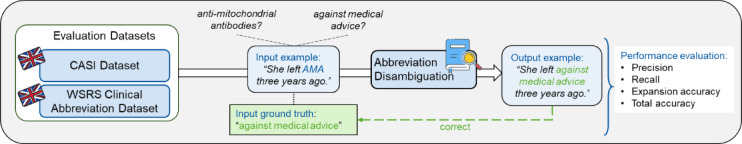
Overview of the abbreviation disambiguation evaluation. The abbreviation disambiguation step was evaluated on the external CASI and WSRS Clinical Abbreviation datasets with the performance metrics precision, recall, expansion accuracy, and total accuracy. Individual data points of both datasets consisting of phrases with abbreviations are disambiguated by the pipeline. The output phrase with an expanded abbreviation is checked against the ground truth from the datasets to compute the performance metrics.

The performance of our abbreviation disambiguation in English was evaluated on the CASI dataset^17^ and the WSRS Clinical Abbreviation dataset^13^ (Fig. 5). We formed a subset of the CASI dataset for the evaluation to align our experiment with existing research, which resulted in 21,564 text snippets containing 63 unique abbreviations and 119 unique abbreviation expansions^13^.

### Translation of clinical notes

For the translation of our German clinical notes to English, we use the open-source language model *facebook/wmt19-en-de* due to its open source availability and high performance with a BiLingual Evaluation Understudy (BLEU) score of 40.8^62^. Clinical notes were divided into smaller chunks for the translation to address input restrictions regarding the maximum number of processable tokens of the language model. After translation, the concatenated chunks form the translated clinical note. The translation step is not evaluated separately, as we did not modify the translation model and refer to its published performance^62^.

### Medical entity linking

#### Preparation of terminology data

To prepare our entity linking approach with the English clinical notes, we parsed the terminologies SNOMED-CT and UMLS. For each concept in the terminologies, we gathered the concept descriptions, such as the synonyms as well as the identifier and category in a concept database. To facilitate fast lookup for similar concept descriptions for a given text portion, we embedded and stored all concept descriptions as vectors using respective SS-LMs. Different concepts, e.g. with different identifiers, hierarchy positions and relations, can be associated through the same description, e.g. synonym, resulting in around 40% of concepts in the MedMentions dataset requiring disambiguation^14^. We solve this challenge analogous to the abbreviation disambiguation by leveraging aggregated context samples for each ambiguous concept description. To ensure comparability of our results with published MedCAT results, we use the UMLS 2018AB release. For the entity linking in our German clinical notes to SNOMED-CT, we use the SNOMED-CT International May 2023 release.

#### SNOMED-CT concept candidate identification

**Fig. 6:**
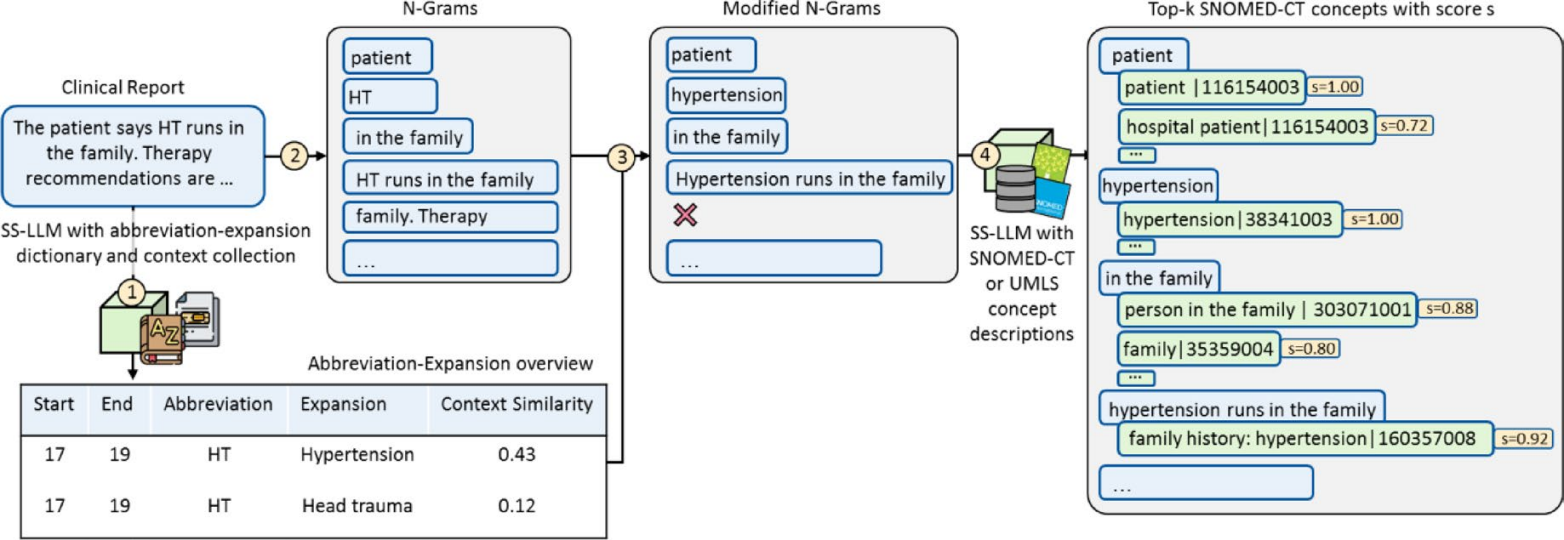
Overview of candidate identification steps. The candidate identification in the entity linking process contains four steps. In the first step in this example, the abbreviation “HT” in the clinical note is identified through pattern matching and disambiguated based on its surrounding context. In the second step, n-grams such as “HT runs in the family” are formed. In the third step, abbreviations are expanded in each n-gram based on the abbreviation-expansion overview, resulting in modified n-grams such as “Hypertension runs in the family”. N-grams such as “family. Therapy” are dropped since a forbidden infix, a full stop, is contained. In the fourth step, for each n-gram the top-k most similar SNOMED-CT descriptions with respective concept identifiers are determined through the language model pre-trained for semantic similarity (SS-LM).

The SNOMED-CT concept candidate identification in the entity linking process contains four steps (Fig. 6).

First, our pipeline identifies known abbreviations from the abbreviation-expansion dictionary and stores positions and expansions of the abbreviations in an abbreviation overview table. The longest abbreviations are matched first to prevent the premature expansion of nested abbreviations.

Second, the clinical note is tokenized by splitting up strings surrounded by non-alphanumeric characters to form n-grams, terms of sequentially occurring n adjacent tokens, such as the 2-gram “the patient”.

Third, it removes terms containing prohibited infixes, which can be defined per use case. In our use case, full stops or hyphens followed by a whitespace as infixes are prohibited since they can indicate a term spanning two sentences or bullet points, leading possibly to erroneous entity links. Abbreviations in the remaining n-grams are expanded based on the table created in the first step. The expansion at this stage ensures that the start and end positions of terms can be stored before modifications, which keeps the stored positions aligned with the positions from evaluation datasets.

Fourth, we retrieve the top-k most similar SNOMED-CT concept descriptions for each n-gram based on the cosine similarity of the respective SS-LM embedding. Retrieving multiple candidates improves recall potential, as the correct candidate, according to ground truth annotations, may not always be the top-ranked match.

#### SNOMED-CT concept candidate selection

**Fig. 7:**
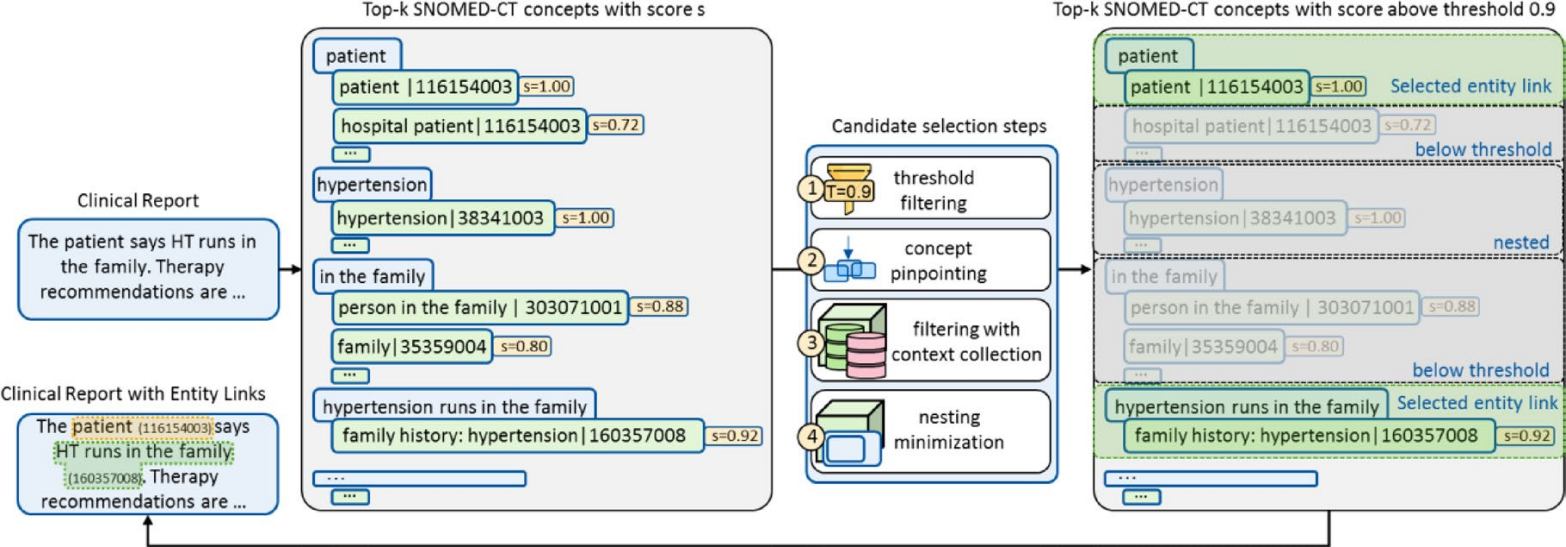
Overview of candidate selection steps. The candidate selection process consists of four sequential steps to refine entity links by removing obsolete and redundant candidates, ensuring only the most accurate links are retained. (1) threshold filtering: Concepts with similarity scores below a predefined threshold, e.g., 0.9, are discarded. For example, the n-gram “patient” originally links to both “hospital patient” and “patient,” but only “patient” is retained as its similarity score of 1.0 exceeds the threshold. (2) concept pinpointing: if multiple n-grams link to the same concept, only the most representative one is kept. For instance, both “The patient” and “patient” may link to “patient | 116154003”, but only “patient” is retained. (3) filtering with context-collection: concepts are further refined based on surrounding contextual information. (4) nesting minimization: entity links containing semantically nested concepts are removed. For example, “hypertension | 38341003” is nested within “family history: hypertension | 160357008” and is therefore eliminated.

The SNOMED-CT concept candidate selection consists of four steps to reduce redundancy and select the most accurate concepts for the entity links from the previously generated candidates. This procedure enables the omission of redundant and obsolete concepts while adapting to data corresponding to specific use cases via iterative online learning. The process uses the entity link candidates from the clinical note of the previous step as input (Fig. 7).

The first step removes any entity links with a cosine similarity below a defined similarity threshold, ensuring that only highly similar concepts are linked to the spans in the text. The second step pinpoints the same concepts, which are linked in several overlapping n-grams with varying similarity to the n-gram with the highest similarity while the other links are removed. The third step removes obsolete concepts through a collection of positive and negative context samples for concepts along the SS-LM, to resolve redundancy in entity links of ambiguous concept descriptions and enable online learning for improved performance. We use the aggregated context from clinical notes in MIMIC-III for ambiguous concept descriptions, which link to more than one concept in the target terminology. These context samples serve as positive context and are supplemented during the entity linking. Negative context is provided through instances where our pipeline incorrectly linked a free-text portion to a concept that was not linked in the ground truth dataset. If a concept has only negative context samples, the pipeline removes entity links containing that concept to avoid repeated incorrect links. Any concepts linked in the ground truth dataset are added to the positive context collection, alongside their surrounding context. If a concept in an entity link has both positive and negative context samples, it is dismissed if the highest negative context similarity exceeds the highest positive context similarity in the current context. The risk of data leakage is addressed by ensuring processing of the training dataset prior before processing the test dataset and only adding context samples after the respective entity linking process. The evaluation of entity links computed after processing a clinical note can improve performance in an online-learning fashion and tailor our pipeline automatically to specific datasets or use-cases.

The fourth step minimizes nesting wherever possible. Our pipeline analyzes whether semantic nesting is present or whether nesting is justified due to different types of information being linked. The pipeline evaluates how omitting the nested n-gram changes the similarity of the outer entity link to its target concept. If an outer n-gram without a nested n-gram yields a lower similarity to the target concept, it indicates that the nested n-gram is semantically nested information. Therefore, the nested entity link is dismissed. An increased similarity instead would indicate no nesting and the presence of new information in the entity link, justifying the nested entity links to prevent information loss.

### Validation

**Fig. 8:**
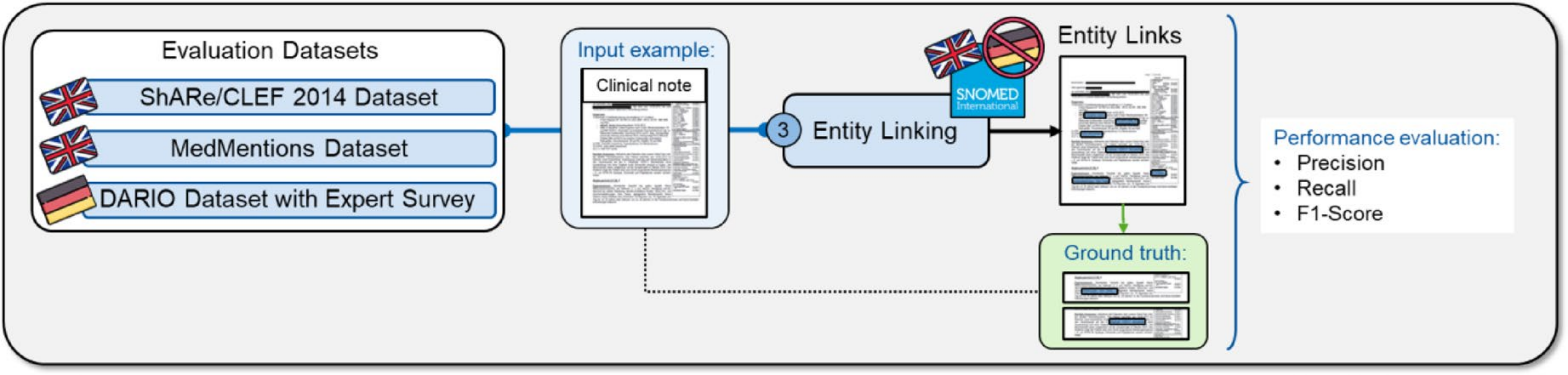
Overview of the evaluation of the entity linking step. The entity linking step was evaluated on the external datasets ShARe/CLEF 2014, MedMentions, and our internal DARIO dataset with the performance metrics recall, precision, and F1-score. In this example, a clinical note is an input for the entity linking step, which then links medical entities in the free text to SNOMED-CT concepts. The entity linking performance is evaluated through the performance metrics by comparing the output with the linked entities in the ground truth.

We evaluated the entity linking performance in English and German clinical notes using three different datasets (Fig. 8). For English, we validated our method on the established MedMentions and ShARe/CLEF 2014 datasets with the target terminologies UMLS and SNOMED-CT and performance metrics recall, precision, and F1-score (Eqs. (1), (2), (5)). For German, we assessed the entity linking performance on the DARIO dataset with the target terminology SNOMED-CT through an expert survey involving three cardiologists.

#### MedMentions

MedMentions consists of over 4,000 abstracts of PubMed publications with more than 350,000 manually linked entities^19^. Mentions of medical entities in the abstracts were linked by professional annotators to concepts of the UMLS 2017AA version, which contains around 3.2 million unique concepts^19^. For our evaluation, we used the pre-defined “disorders only” subset as outlined in the dataset description to focus on health-related concepts and omit other biomedical concepts.

#### ShARe/CLEF 2014

ShARe/CLEF 2014 comprises 433 de-identified clinical notes from over 30,000 patients admitted to the intensive-care unit (ICU), which were annotated by 15 clinical professionals and informatics experts^63^. We use the development set containing 300 documents of four different types: discharge summary, radiology report, electrocardiogram and echocardiogram. The annotations consist of concepts of the UMLS version 2011AA belonging to the “Disorder” semantic group and that are linked to SNOMED-CT^18^. To evaluate our entity linking approach, we only retained annotations linking text spans to UMLS identifiers, excluding “CUI-less” annotations, which are annotations without unique concept identifier (CUI), that make up around 30% of th dataset^64,65^.

We evaluated the entity linking performance of our pipeline by measuring the recall after candidate identification and the recall, precision and F1 score after the candidate selection.

An entity link is considered correct only if it matches the exact text portion and concept identifier as the ground truth annotations. The entity linking was evaluated across high similarity thresholds ranging from 0.9 to 0.99 to avoid degrading the performance through linking text portion to semantically less dissimilar concepts and keep resource-intensive computations low. To evaluate the performance gain in our pipeline with the abbreviation disambiguation step, we also evaluated the performance difference in our approach by disambiguating abbreviations in the clinical notes prior to the entity linking step.

#### DARIO dataset

To evaluate the entity linking performance on German clinical notes, we used patient data from our internal heart failure cohort dataset (DARIO dataset). The DARIO dataset comprises retrospective health records from 2012 to 2016 for 846 patients from the Department of Internal Medicine I at the University Hospital Aachen, with 351 patients with no heart failure (41.5%), 193 patients with HFrEF (22.8%), 175 patients with HFmrEF (20.7) and 127 patients with HFpEF (15%). The distribution of the DARIO cohort reflects the real-world prevalence of heart failure subtypes in specialized clinical settings. Our retrospective study was approved by the Ethics Committee of the Medical Faculty at RWTH Aachen University (EK 416 − 21). The patient data includes structured EHR data containing demographic information, diagnoses, conditions, and laboratory values as obtained from blood tests or electrocardiography in a tabular format and unstructured data as clinical free-text notes in the form of discharge summaries. The clinical notes cover different medical aspects such as the diagnosis, cardiac history, family history, social history, patient progression, procedure and recommended therapy of the patient. Additionally, the clinical note may contain a report sheet with examination findings and other comments. Patients can be diagnosed with heart failure (HF) and differentiated into three subtypes based on the ejection fraction (EF): reduced (HFrEF), mildly reduced (HFmrEF) or preserved (HFpEF).

Since the DARIO dataset lacks concept links to SNOMED-CT or UMLS, we cannot automatically evaluate linked concepts against ground truth annotations. Instead, we evaluated the goodness of the linked entities by our entity linking approach from the German clinical notes of the DARIO dataset through an expert survey conducted with three cardiologists from the Department of Internal Medicine I of the University Hospital Aachen. The survey questions consisted of 120 randomly sampled entity links of the SNOMED-CT categories “Body structure”, “Procedures” and “Clinical finding”. Each question contained a text snippet, in which a portion of the text corresponding to a linked SNOMED-CT concept was highlighted and the SNOMED-CT concept was listed with its corresponding identifier. The physicians were asked to rate randomly-sampled linked entities on a 3-Point LIKERT scale from “no match”, “partial match” and “exact match”. Only SNOMED-CT concepts of the three categories “Procedures”, “Body Structures” and “Clinical Findings” were evaluated to ensure feasibility of the expert evaluation. Linked entities are sampled in a stratified manner based on heart failure classification and the corresponding SNOMED-CT hierarchy. In total, 120 entity links are sampled, with 30 entity links per heart failure class, including 10 entity links for each of the three SNOMED-CT hierarchies. The survey consists of three questionnaires, each containing 40 entity links of the same SNOMED-CT concept category across all four heart failure patient classes. The inter-annotator agreement (IAA) of the physicians is evaluated using the Cohen’s Kappa score^48^, with values ranging from − 1 to 1 to indicate chance agreement with low values up to strong agreement with high values.

### Heart failure diagnosis

For heart failure prediction our novel pipeline uses a Support Vector Machine (SVM) classifier, which was trained with patient data of the DARIO dataset. We focused on diagnostic support of heart failure of hospital patients, to reduce frequent misdiagnoses of patients and evaluate our novel comprehensive pipeline to serve in future use cases with patient data of early disease stages, such as clinical notes of general practitioners.

**Fig. 9:**
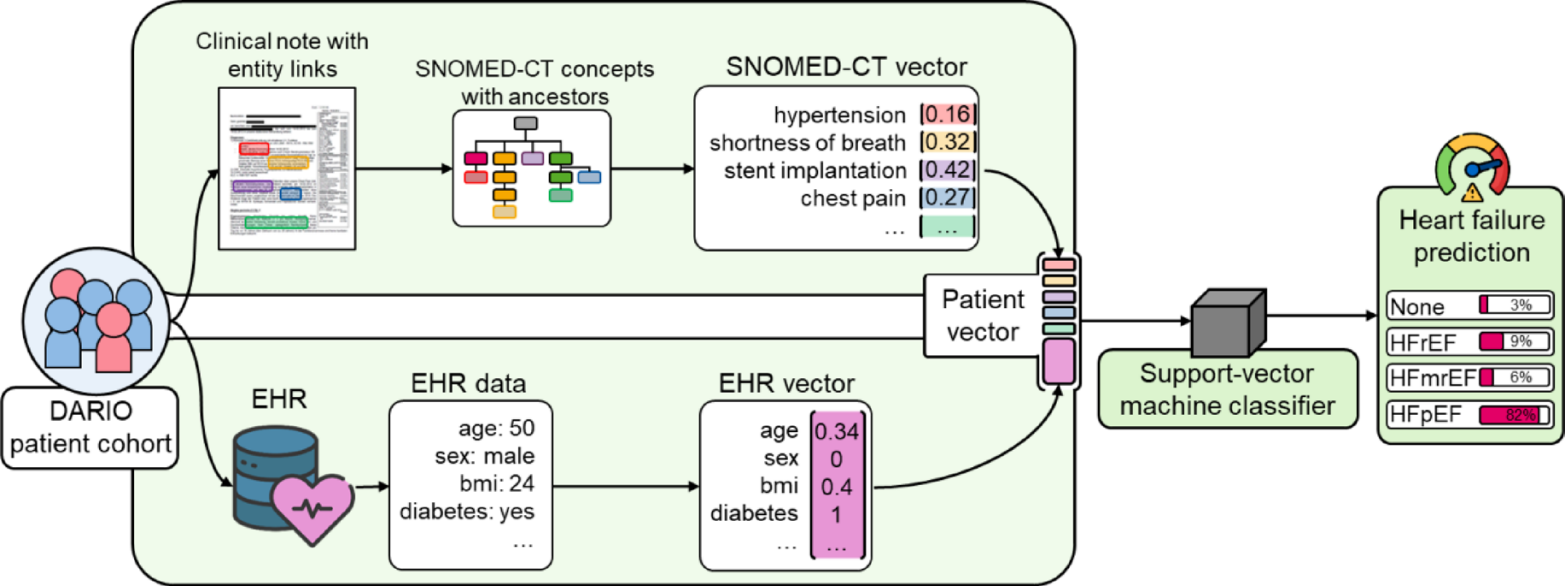
Overview of the classification approach. The classification step leverages patient data in the form of clinical notes with entity links and electronic health records (EHR). Concepts from entity links, such as hypertension or chest pain, alongside corresponding SNOMED-CT ancestor concepts make up a weighted SNOMED-CT vector for each patient. Each patient’s SNOMED-CT vector is combined and standardized with a vector containing their EHR data, such as their age or BMI, to form a patient vector. Patient vectors are used for training and testing of the Support-vector machine classifier, which predicts a heart failure diagnosis for each patient – in this case with an 82% probability of the patient suffering from HFpEF.

The classifier is trained with patient data in form of EHR and obtained SNOMED-CT concepts from the previously computed entity links to compute heart failure predictions for each patient (Fig. 9).

In the classification step of our pipeline, data of patients from the DARIO project cohort in form of clinical notes and EHR data is processed separately. Information in clinical notes is linked to SNOMED-CT concepts, for which all associated ancestor concepts are retrieved and assigned a weight based on the term frequency-inverted document frequency (TF-IDF) weighting scheme. We use the scikit-learn TF-IDF transformer fit on the training dataset to assign weights to each SNOMED-CT concept based on its frequency in a clinical note in relation to the frequency across the clinical notes of all patients, similar to prior research assigning TF-IDF weights to keywords of radiological reports^66^. The EHR data underwent data cleaning to address missing values and inconsistencies, followed by normalization to construct each patient’s EHR vector. The resulting SNOMED-CT vector and EHR vector are combined to represent each patient with a comprehensive vector, which serves as input for the SVM classifier for heart failure prediction. Since the TF-IDF transformer is fit on the training data only to prevent data leakage, the SNOMED-CT vector is sparse when encoding SNOMED-CT concepts per patient in the test dataset. We chose an SVM classifier due to high performance when facing the sparsity and high dimensionality of the SNOMED-CT vector. The SVM classifier is then trained with patient vectors to predict a heart failure risk for each of the four heart failure groups of patients having no heart failure (None), heart failure (HF) with reduced ejection fraction (HFrEF), mildly reduced ejection fraction (HFmrEF) or preserved ejection fraction (HFpEF). For the SVM implementation we used the scikit-learn SGDClassifier and modified the default parameters with the ‘hinge’ loss to train a SVM with a fixed random_state of ‘2026’ and the class_weight set as ‘balanced’ to account for the dataset imbalance.

To assess the extent to which heart failure classification can be improved by using data extracted from clinical notes, we compared the performance of three classifiers: one trained solely on entity link data, another on tabular data in the form of EHR data, and a classifier trained on both types of data.

All classifications took place with stratified 5-fold cross-validation across all four heart failure classes and with a fixed random seed ‘2026’ for reproducibility.

Moreover, to validate the performance gain of our implemented pipeline, we compared the performance to a fine-tuned medBERT.de model for the classification task, which directly produces a heart failure prediction based on clinical notes without requiring entity linking to SNOMED-CT concepts. medBERT.de is a novel comprehensive German BERT model trained on 4.7 million diverse medical documents and achieved state-of-the-art performance on multiple medical benchmarks. medBERT.de poses a neural baseline with two classification approaches: first, the classification is performed solely on clinical notes and second, through late-fusion clinical notes and EHR are combined for a comprehensive diagnosis. For the late-fusion approach, we implemented a multimodal neural architecture extending the medBERT.de classification approach. The text data is processed via the medBERT.de transformer to extract a semantic embedding classify token (CLS token), while the structured EHR data is processed through a parallel Multi-Layer Perceptron (MLP) branch consisting of two linear layers with Rectified Linear Unit (ReLU) activation and dropout. These two representations are then concatenated to form a joint feature vector, which serves as the input to a final classification head. This architecture allows to simultaneously optimize the text embeddings and the tabular feature weights during the training process to identify cross-modal correlations. The medBERT.de text-based and multimodal approach with the MLP were configured with a learning rate of 2e-5, evaluation strategy set to ‘epoch’ with a maximum of 10 epochs, early stopping patience set to 3 and early stopping threshold of 0.001 to allow up to three consecutive training iterations without performance improvement before stopping the training process. The optimization took place towards the ‘f1’ score with a fixed random seed set to ‘2026’.

## Data Availability

The UMLS 2018AB release is provided by the National Library of Medicine and requires an UMLS Metathesaurus License: https://www.nlm.nih.gov/research/umls/licensedcontent/umlsarchives04.html. The SNOMED-CT International releases provided by SNOMED International require a SNOMED-CT license and access to the Member Licensing and Distribution Service (MLDS). Since monthly releases are only retained for a year, the SNOMED-CT International May 2023 release we used is not available as a download, but can be accessed in the SNOMED CT Browser here: http://snomed.info/sct/900000000000207008/version/20230531. The MIMIC-III dataset is provided by PhysioNet here: https://physionet.org/content/mimiciii/1.4. The GGPONC dataset is provided by the Hasso Plattner Institute and can be acquired here: https://www.leitlinienprogramm-onkologie.de/projekte/ggponc-english. The CASI dataset is provided by the University of Minnesota here: https://conservancy.umn.edu/items/6651323b-444a-479e-a41a-abca58c2e721. The dataset used for the second validation of our abbreviation disambiguation, referred to in this paper as the ”WSRS Clinical Abbreviation Dataset” for clarity, was originally used in “Deciphering clinical abbreviations with a privacy protecting machine learning system”^13^, where steps to gain access are also outlined in the “Data Availability section”: https://www.nature.com/articles/s41467-022-35007-9. The MedMentions dataset is provided here: https://github.com/chanzuckerberg/MedMentions. The ShARE/CLEF 2014 dataset is provided by PhysioNet here: https://physionet.org/content/shareclefehealth2014task2/1.0. The German dataset of the DARIO research project used in this study cannot be publicly shared due to patient privacy concerns.The used language models are openly available on Hugging Face (https://huggingface.co/) and can be found for *BioLORD* under https://huggingface.co/FremyCompany/BioLORD-2023 and for *paraphrase-multilingual-MiniLM-L12-v2* under https://huggingface.co/sentence-transformers/paraphrase-multilingual-MiniLM-L12-v2.

## Code availability

The code developed in this study is property of the University Hospital Aachen and cannot be publicly shared.
